# A rapid yolk-sac serum extraction method for quantifying maternal IgY of individual birds^☆^

**DOI:** 10.1016/j.mex.2026.104027

**Published:** 2026-07-03

**Authors:** Anna L.F.V. Assumpcao, Tanmaie Kalapala, Geetha Kumar-Phillips, Palmy R.R. Jesudhasan, Komala Arsi

**Affiliations:** a Department of Poultry Science, University of Arkansas, Fayetteville, AR, United States; b Poultry Production and Product Safety Research Unit, ARS, USDA, Fayetteville, AR, United States

**Keywords:** Maternal immunity, Antibody, ELISA, Chicken, Poultry

## Abstract

Maternal immunoglobulin Y (IgY) antibodies in birds are deposited into the egg yolk and are later transferred to the developing embryo via the yolk sac during embryonic development. Quantification of IgY in egg yolk and yolk sac sera is used to assess maternal antibody transfer. Current yolk sac serum extraction methods require large sample volumes, necessitating the pooling of yolk sac samples from several birds. Here, we describe a modified yolk-sac serum extraction protocol that requires a smaller volume and enables individual quantification of IgY, allowing assessment of inter-individual variability in maternal antibody transfer within a flock. The proposed method, while using a reduced sample volume, also maintains antibody levels for downstream immunoassays. While pooled samples provide an overview of the flock's immunological status, analyzing individual antibody levels enables assessment of variability in vertical antibody transfer, which may reflect differences in maternal immune status.

• Individual maternal antibody transfer quantification.

• Small sample volume input.

• Rapid and efficient method for preparing samples compatible with immunoassays.

## Graphical abstract

“A rapid yolk-sac serum extraction method for quantifying maternal IgY of individual birds.” created in BioRender.com. Assumpcao, ALFV (2026) https://app.biorender.com/illustrations.

**Figure:**
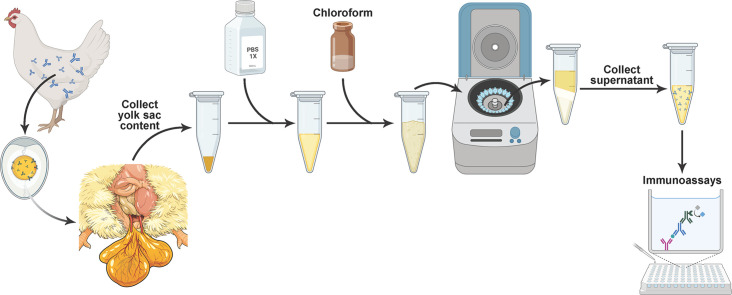

Specifications table

**Table:** 

**Subject area**	Immunology and Microbiology
**More specific subject area**	Avian Immunology; maternal antibody transference; IgY quantification
**Name of your method**	Individual yolk sac serum extraction for IgY quantification method
**Name and reference of original method**	[1] K.R. Hamal, S.C. Burgess, I.Y. Pevzner, G.F. Erf, Maternal antibody transfer from dams to their egg yolks, egg whites, and chicks in meat lines of chickens, Poult Sci 85(8) (2006) 1364–72.
**Resource availability**	Chloroform, Dulbecco's phosphate-buffered saline (D-PBS), Abcam Chicken IgY ELISA kit.

## Background

In avian species, the yolk sac tissue is a multifunctional organ responsible for host immunity, erythropoiesis, nutrient uptake, carbohydrate and lipid metabolism during embryonic stages. During egg formation, maternal antibodies are transferred from hen to egg yolk by deposition of immunoglobulin Y (IgY). The yolk sac tissue transports maternal antibodies from the yolk into the embryonic bloodstream to provide passive immunity to the embryo through selective transport [[2], [3], [4]]. The quantification of maternal IgY in egg yolk and yolk sac is commonly used as an indicator of vertical antibody transfer and of the flock’s early-life infection protection status. Quantification of maternal antibody transfer can reflect the immunological status of breeder hens [1,5]. The investigation of vertical passive immunity transfer is important for vaccine development and understanding pathogen-specific antibody transfer in poultry production and experimental avian systems.

The term “IgY technology” refers to a procedure in which birds, more specifically laying hens, are immunized to produce polyclonal IgY antibodies against specific targets. These antibodies are subsequently isolated in large quantities from egg yolk and applied in diverse biotechnology and biomedical contexts [[6], [7], [8], [9]]. IgY-based assays have been increasingly used for their high specificity, reduced cross-reactivity with mammalian immune components, and compatibility with downstream immunological analyses such as enzyme-linked immunosorbent assays (ELISAs) [8,9]. A significant number of protocols for avian IgY extraction and purification have been developed to isolate IgY from egg yolk, including water dilution, polyethylene glycol (PEG) precipitation, ammonium sulfate precipitation, and solvent-based lipid removal methods [[10], [11], [12]]. Most of these techniques are designed for egg yolk samples and require pooling of samples to obtain sufficient volume for effective serum extraction. The selection of an appropriate method is determined by the intended application, desired purity, and available resources [10,11].

Although not common, methods for extracting yolk sac serum from day-of-hatch chicks are available. However, these methods usually require a large sample volume [5], necessitating pooling, or are time-consuming and complicated [12]. Although pooled samples enable us to evaluate the overall flock immunity, individual variances are lost in the process. Evaluating differences in antibody transfer among individual chicks is necessary to understand the physiological mechanism underlying maternal antibody transfer. Additionally, these differences can provide information about the hen’s immune health, vaccine response, or egg traits among breeders. Methods with multiple processing steps and extended overnight incubations, which increase total processing time, decrease overall efficiency, and increase the likelihood of technical errors. To address these limitations, we developed a rapid, simplified yolk sac serum extraction method that uses a smaller sample volume, adapted from Hamal et al. method for yolk serum extraction [1]. This method allows measurement of maternal antibodies in individual birds, aiding the study of differences in antibody transfer within bird populations.

## Method details

**Fig. 1:**
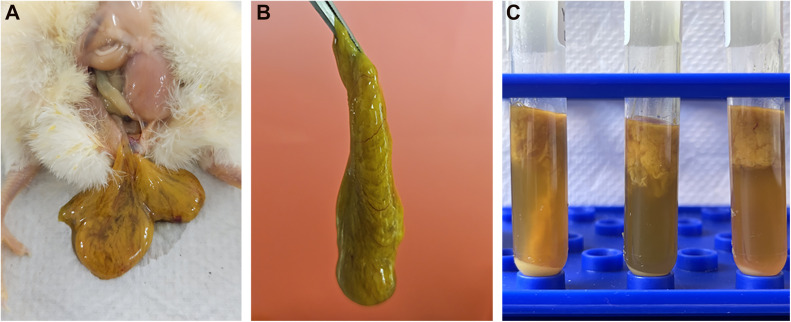
Collecting yolk sac sample. (A) Yolk sac isolated from other abdominal organs. (B) Yolk sac. (C) Yolk sac in a sterile tube after overnight refrigeration.
○ Isolated the yolk sac (Fig. 1B)
○ Transferred it into a sterile capped tube (Fig. 1C)
○ Kept refrigerated until start of serum extraction

Reagents:

• Dulbecco's phosphate-buffered saline (1 × PSB)
• Chloroform

1. Sample collection:

○ Euthanized one-day-old chicken (hatchling)
○ Using scissors, opened the abdominal cavity (Fig. 1A)

**Fig. 2:**
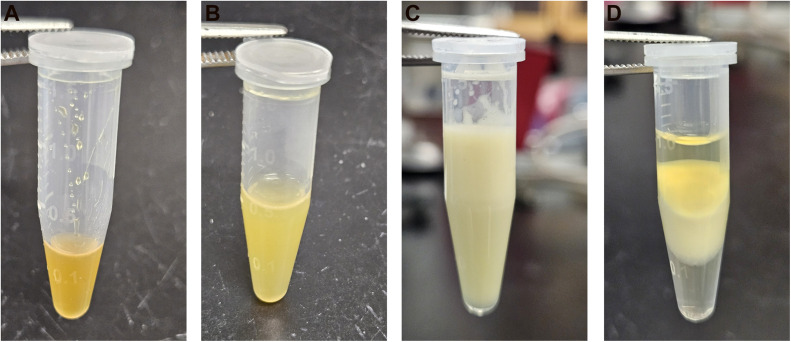
Steps of yolk sac sera extraction. (A) Yolk sac content. (B) Yolk sac content mixed with 1 × PBS. (C) Sample after adding chloroform and vortexing. (D) Sample after centrifugation showing the 3 layers - Top: yellow, clear or slightly turbid (supernatant); Middle: milky white and highly turbid; Bottom: clear with mild turbidity.

2. Serum Extraction:

○ Transferred 200 µL of yolk sac content into a pre-labeled tube (Fig. 2A)

○ Added 400 µL of 1 × PBS

○ Mixed the tube contents well by shaking (Fig. 2B)

○ Added 600 µL of Chloroform to the tube

○ Vortexed the tube for 30 s (Fig. 2C)

○ Centrifuged at 1750 × g for 30 min at room temperature

○ Samples should show 3 layers – Top: yellow, clear or slightly turbid (supernatant); Middle: milky white and highly turbid; Bottom: clear with mild turbidity (Fig. 2D)

○ Collected top layer supernatant (sample is diluted 1:3)

○ Samples can either be used directly for an immunoassay protocol or stored at −80 °C

## Method validation

**Fig. 3:**
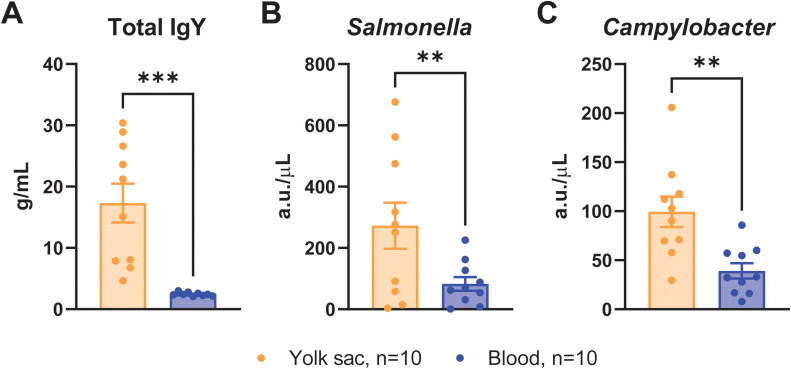
Validation of the method using immunoassays. (A) Results of the ELISA kit assay quantifying total IgY from chicken yolk sac and blood sera. (B) Indirect ELISA assay quantification of anti-*Salmonella enterica* IgY in chicken yolk sac and blood sera. (C) Indirect ELISA assay results for the quantification of anti-*Campylobacter jejuni* IgY in chicken yolk sac and blood sera. N represents the number of chickens evaluated; graphs show means ± SEM. **p-value <0.01; ***p-value < 0.005.

To establish the validity of this protocol, we evaluated yolk sac samples along with the blood serum samples from 10 birds on the day of hatch. To first validate this method, total IgY levels in ten samples of extracted yolk sac serum were determined using the IgY ELISA kit (Abcam, Catalog # ab157693). The mean total IgY concentration in yolk sac samples was 18.73 g/mL ± 3.164 g/mL (Fig. 3A). Furthermore, we quantified the IgY concentration in blood serum samples from the same birds (mean 2.45 g/mL ± 0.097 g/mL), which presented less variability than the extracted yolk sac serum (Fig. 3A). The observed variability in the yolk sac samples could be associated with the differences in the yolk sac size among birds. Standardizing measurements by weighing each yolk sac and normalizing IgY levels to total yolk sac mass (g of IgY per yolk sac) could reduce variability between samples and improve the accuracy of comparisons. Comparing both sample types by a paired *t*-test, we concluded that the extracted yolk sac serum showed significantly higher IgY levels than blood serum samples (p-value = 0.0009; Fig. 3A).

To further validate the methods, we measured the anti-*Salmonella* and anti-*Campylobacter* IgY levels in yolk sac and blood sera from the same 10 birds on the day of hatch using an ELISA protocol adapted from Assumpcao et al. [13]. For the anti-*Salmonella* IgY levels, ELISA plates were coated with five relevant *S. enterica* serovars. The extracted yolk sac serum samples had over 3-fold higher levels (mean = 272.7 a.u./µL) of anti-*Salmonella* IgY than blood serum samples (mean = 82.67 a.u./µL). The paired *t*-test showed a significant difference between the two sample types, p-value = 0.0077 (Fig. 3B). Similarly, to evaluate IgY against *C. jejuni*, ELISA plates were coated with seven wild *C. jejuni* strains. Extracted yolk sac sera (mean = 99.41 a.u./µL) showed >2.5-fold increased anti-*C. jejuni* IgY compared to blood sera (mean = 39.14 a.u./µL). As observed with anti-Salmonella IgY, the paired *t*-test showed a significant difference between the sample types (p-value = 0.0038; Fig. 3C). These results confirm that this method effectively extracts samples for measuring both total and antigen-specific IgY antibodies, supporting its suitability for downstream immunological applications.

Our results demonstrate that the yolk sac serum extraction method generates samples that can be consistently used to measure total and antigen-specific IgY, supporting its reliability for immunological assays. Total IgY levels were found to be 8-fold greater than in yolk sac sera compared to blood sera, while IgY levels against *S. enterica* and *C. jejuni* were only 2.5 to 3-fold higher when compared to blood sera from the same birds. This suggests that maternal immunity transfer rates may differ by pathogen and that yolk sac serum provides a more detailed assessment of this transfer in day-of-hatch chicks than blood serum. Previous studies showed that chicks of older breeders may have a higher IgY absorption rate compared to younger hens [1,5]. These results suggest that unknown factors interfere with IgY transfer from the yolk sac to chicks, and further research is needed to elucidate the physiological mechanisms underlying these processes.

The proposed chicken yolk sac serum extraction method has several advantages over conventional approaches [1,10,12,14] as it minimizes the required sample volume and is a fast extraction technique that yields reliable results (Table 1). Smaller sample volumes enable processing of individual samples without pooling, thereby enabling detection of inter-individual variability in maternal antibody transfer. This increased analytical resolution at the individual-embryo level is important for evaluating maternal immune transfer, which is relevant to developing vaccines and understanding early-life protection, as individual-level differences in antibody deposition may influence offspring health. Furthermore, this method is designed to be simple and reproducible using commonly available reagents and standard equipment, compared with previously published methods [12]. Collectively, these findings demonstrate the usefulness and sensitivity of the proposed method for applications that require reliable quantification of yolk sac-derived antibodies from limited sample volumes.

**Table 1:** Comparison of the described method with the adapted method and other yolk sac serum extraction protocols.

	Our method	Hamal et al. [1]	Ulmer-Franco et al. [5]	Kaspers et al. [12]
**Sample extracted**	Yolk sac	Yolk	Yolk sac	Yolk sac
**Preparation before collecting samples**	-	-	-	Tracer-labeled IgA was injected into the albumen before egg incubation
**Storage of the sample**	Proceed directly to extraction or refrigerated (up to 48 h)	Proceed directly to extraction	Store at −20 °C	Yolk sac sample was diluted with PBS and stored at −20 °C
**Equipment necessary**	Microcentrifuge Chemical safety hood (to handle chloroform)	Centrifuge Chemical safety hood (to handle chloroform)	Refrigerator (for overnight incubation) Refrigerated centrifuge	Radiolabel reagents, chromatography equipment, and reagents. Dextransulfate precipitation method and gel filtration reagents.
**Volume required for extraction**	0.2 mL	All yolk volume (approximately 10–15 mL)	150–200 mg	^125^I-IgA labeled required.
**Time for extraction**	Approximately 1 h	Approximately 1 h	>16 h (overnight incubation)	>48 h
**Centrifugation speed and time**	1750 × g for 30 min	1000 × g for 30 min	10,062 × g for 15 min	No information.
**Immunoglobulin tested**	IgY	IgY	IgY	IgA
**Immunoglobulin concentration**	18.73 ± 3.164 g/mL	22.5 and 43.9 mg/yolk	Approximately 60 to 90 mg/yolk sac	240.2 ± 83.5 µg/mL

## Limitations

This method should be applied to yolk sac samples that were refrigerated for <72 h. The method was not validated on frozen samples.

## Ethics statements

All procedures were approved by the University of Arkansas Institutional Animal Care and Use Committee (Protocol#24,041 and 25,012) before the study was initiated.

## CRediT authorship contribution statement

**Anna L.F.V. Assumpcao:** Conceptualization, Formal analysis, Investigation, Methodology, Supervision, Validation, Visualization, Writing – original draft. **Tanmaie Kalapala:** Investigation, Methodology, Writing – review & editing. **Geetha Kumar-Phillips:** Validation, Writing – review & editing. **Palmy R.R. Jesudhasan:** Resources, Writing – review & editing. **Komala Arsi:** Conceptualization, Funding acquisition, Resources, Supervision, Writing – review & editing.

## Declaration of interests

The authors declare that they have no known competing financial interests or personal relationships that could have appeared to influence the work reported in this paper.

## Data Availability

Data will be made available on request.
